# Public Beliefs and Attitudes towards Depression in Italy: A National Survey

**DOI:** 10.1371/journal.pone.0063806

**Published:** 2013-05-20

**Authors:** Carmine Munizza, Piergiorgio Argentero, Alessandro Coppo, Giuseppe Tibaldi, Massimo Di Giannantonio, Rocco Luigi Picci, Paola Rucci

**Affiliations:** 1 Centro Studi e Ricerche in Psichiatria, Torino, Italy; 2 Dipartimento di Psicologia Università di Pavia, Pavia, Italy; 3 Facoltà di Psicologia, Università di Chieti, Chieti, Italy; 4 Facoltà di Medicina e Chirurgia, Università di Torino, Torino, Italy; 5 Dipartimento di Scienze Biomediche e Neuromotorie, Alma Mater Studiorum Università di Bologna, Bologna, Italy; Federal University of Rio de Janeiro, Brazil

## Abstract

**Background:**

Previous studies have shown that attitudes towards depression may be influenced by country-specific social and cultural factors. A survey was carried out to collect beliefs on and attitudes toward depression in Italy, which has an established community-based mental health system.

**Methods:**

A telephone survey was carried out in a probabilistic sample aged ≥15 years. A 20-item questionnaire was administered to explore knowledge of depression, stigma, causal beliefs, treatment preference, and help-seeking attitudes.

**Results:**

Of the 1001 participants, 98% were aware of depression, and 62% had experienced it, either directly or indirectly. A widespread belief (75%) was that people suffering from depression should avoid talking about their problem. A minority of the sample viewed depression as a condition that should be managed without recourse to external help or a “socially dangerous” illness. Among perceived causes of depression, most respondents mentioned life stressors or physical strains. Psychologists were often indicated as an adequate source of professional help. Half of the sample believed that depression should be pharmacologically treated, but drugs were often seen as addictive. Referring to a primary care physician (PCP) was considered embarrassing; furthermore, many people thought that PCPs are too busy to treat patients suffering from depression.

**Conclusions:**

Our findings indicate that depression is seen as a reaction to significant life events that should be overcome with the support of significant others or the help of health professionals (mainly psychologists). However, there are still barriers to the disclosure of depressive symptoms to PCPs, and concerns about the addictive effect of antidepressants. In the presence of a gap between people’s beliefs and what health professionals consider appropriate for the treatment of depression, a “shared decision making” approach to treatment selection should be adopted taking into account the patients’ preference for psychological interventions to ensure active compliance with effective treatments.

## Introduction

Major depression is a widespread mental disorder. Evidence from the European Study of the Epidemiology of Mental Disorders [1] indicates that the lifetime prevalence of mood disorders (depression and dysthymia) across 6 European countries is 14%, and the 12-month prevalence 4.2%. Italy (11% lifetime, 3% 12-months) has one of the lowest prevalence rates among European countries [2].

The prevalence and incidence of depression vary considerably among countries, suggesting the influence of specific socio-cultural aspects or peculiar risk factors: adult prevalence ranges from 1.5% in Taiwan to 19.0% in Beirut, whereas incidence varies between 0.8% in Taiwan and 5.8% in New Zealand. The average age at onset ranges from 24.8 to 34.8 years [3].

Major depression is twice more frequent in women than in men: this is confirmed by a number of U.S. and European studies [4]–[6], and by most epidemiological surveys in the U.S., such as the NCS Study [7], [8] and the ECA Study [9]. More specifically, lifetime prevalence in women ranges between 10% and 25%, and 5–12% in men [10]; gender differences persist from early adolescence to middle age [7].

In addition to their symptoms, people with depression must cope with the stigma associated with this disorder. According to the definition of Link and Phelan [11], stigma consists of five interrelated components: labeling, stereotyping, separation, status loss, and discrimination.

Prejudice and discrimination are linked to commonly held stereotypes that are associated with mental illness. These stereotypes depict individuals with a mental illness as unable to make competent decisions and dangerous to themselves and/or the others [12]. Among people suffering from depression, self-stigma may greatly interfere with the individual’s decision to seek treatment, resulting in treatment delay or avoidance [13]. In those who do seek treatment, stigma may be partially responsible for non-adherence to treatment regimens [14].

Therefore, although antidepressants and brief structured psychotherapy are effective in 60–80% of people suffering from depression [15], less than one half receive some treatment [16].

In the last two decades a number of international surveys have focused on people’s beliefs and attitudes towards mental illness: these studies promoted a broad debate over personal capacities to cope with depression and possible strategies to improve such skills. Research on this topic has been carried out in Great Britain [17] Australia [18], and Germany [19]. Other surveys were aimed at comparing public beliefs about mental illness in different nations and cultures [20], [21].

Some studies have shown that among people suffering from depression, the propensity to seek help is higher when associated with the ability to recognize the disorder and its causes [18], the ability to identify professionals and forms of treatment that could provide some help, and confidence in the positive outcome of such treatments [22]. Moreover, a strong relationship has also been found between the subjects’ attitudes toward depression and their beliefs on its primary causes. In an Australian national survey [23], respondents using accurate psychiatric labels were more likely to view a person with mental health problems as sick rather than weak.

Our chances to tackle depression and to make the best possible use of available care opportunities could be significantly improved by measuring the extent to which the professionals’ views on depression and their care practices are shared by the general population. One should however bear in mind that many beliefs and attitudes towards the disease are highly sensitive to cultural change.

Since the closure of psychiatric hospitals in 1978, mental health care in Italy is delivered by Mental Health Departments that are in charge of the management and planning of all community-based medical and social activities related to prevention, treatment, and rehabilitation in a defined catchment area. Access to mental health services can be direct or through referral from primary care physicians. Mild to moderate depression is usually treated by primary care physicians, but local care pathways have been implemented in some Italian regions based on collaborative care models to treat effectively the full spectrum of depression. To date, it is unknown whether this established community-based mental health system contributed to reducing the stigma on mental health, or whether it has affected the mental health literacy in the Italian population. In order to examine the state-of-the-art on attitudes and beliefs on mental disorders in Italy, we decided to undertake a population survey with a focus on depression, that is the most common mental disorder.

The purpose of this work consists in surveying beliefs and attitudes toward depression in a representative sample of the Italian general population. The survey was focused on awareness of and stigma on depression, beliefs on its possible causes, treatment preferences and help-seeking attitudes. Specifically we investigated willingness to disclose depressive symptoms to primary care physicians, beliefs on pharmacological treatment and psychotherapy, as well as on coping strategies based on acquiring new habits. Lastly, we investigated the extent of stigma on depression, and its possible influence on how people deal with the illness.

## Methods

### Ethics Statement

The survey was carried out through telephone interview by a survey research organization between 4/29/20102010 and 5/3/2010, using the C.A.T.I. (Computer Assisted Telephone Interview) technique. Telephone surveys on attitudes and beliefs are exempt from approval from the Italian Ethics Committee, but are subject to the law ‘Protection of individuals and other subjects with regard to the processing of personal data, ACT no. 675 of 31.12.1996′ (amended by Legislative Decree no. 123 of 09.05.1997, no. 255 of 28.07.1997, no. 135 of 08.05.1998, no. 171 of 13.05.1998, no. 389 of 6.11.1998, no. 51 of 26.02.1999, no. 135 of 11.05.1999, no. 281 of 30.07.1999, no. 282 of 30.07.1999 and no. 467 of 28.12.2001).The Italian Data Protection Authority (Garante per la protezione dei dati personali) is an independent authority set up to protect fundamental rights and freedom in connection with the processing of personal data, and to ensure respect for individuals' dignity.

Data were collected and analysed in compliance with the Italian “Code of conduct and professional practice applying to processing of personal data for statistical and scientific purposes” enforced by this Authority http://www.garanteprivacy.it/web/guest/home/docweb/-/docweb-display/docweb/1115480 (Published in the Official Journal no. 190 of August 14, 2004).

All participants (including minors) gave their consent to participate in this study. Said consent was documented in compliance with Article 9, section 4b of the code mentioned above.

The main topics to be explored in the interview were determined through a review of the available literature on current attitudes toward depression and possible treatment strategies by searching PUBMED for the years between 1996 and March 2009 using following search string: (recognition OR attitude OR perception OR belief) AND depression AND (public OR people). Only surveys with a clear definition of methods employed were considered eligible and 29 studies were selected.

Based on the literature review, a structured 20-item questionnaire was developed encompassing all the selected topics (see appendix S1). The instrument is organized into four sections: demographic characteristics, attitudes towards depression, choice of treatment, experience of depression.

The specific phrasing of each question and the language to be used during the interview were defined in advance by a focus group formed by psychiatric clinicians and care providers (2 doctors, 2 psychologists, 2 tutors, 2 social workers, 7 nurses), and tested through 10 pilot interviews with randomly chosen subjects.

For the large majority of items, respondents were asked to express their agreement with a statement on a Likert scale ranging from 1 = complete disagreement to 4 = strong agreement or their beliefs from 1 = very likely to 4 = not at all likely. For some items, respondents were asked to choose their answer from a list of possible alternatives. To reduce bias, the order of possible responses to multiple-choice questions was rotated.

For the purpose of the analyses reported in the present paper, responses to questions 8, 10, 12, 14, 15, 16, 17, 18 were dichotomized to reflect agreement or disagreement. For instance, the response to question 10.3: ‘People suffering from depression are dangerous to the others’ was dichotomized into strongly agree/fairly agree vs. fairly somewhat disagree/disagree. Similarly, responses about causes of depression were dichotomized as very likely/quite likely vs. little likely/not at all likely. or. Multiple logistic regression models were used to analyse the relationship of dichotomous responses with age, gender, education and experience of depression. Age was categorized into decades and education was used as a categorical variable with 5 categories: university degree, high school diploma, secondary school diploma, primary school diploma, <5 years of education.

The proportion of people suffering from depression estimated by respondents was analysed as a function of gender, age, and direct experience of depression using analysis of variance (ANOVA) or t-test as appropriate. Following significant ANOVA F-test, pairwise post-hoc comparisons were performed at Bonferroni-adjusted probability level.

Analyses were performed using the Statistical Package for Social Sciences (SPSS), version 20.0.

### Sampling

The target population was the Italian population aged ≥15 years. A stratified, multistage probability sampling was used to get a representative sample of the national census population. The survey was aimed at interviewing about 1000 subjects.

To achieve this goal, a stratified sample of 15.000 families was randomly chosen from the list of 22 million Italian landline telephone subscribers. Strata were the geographical region, the type of municipality (urban/non-urban) and the size of the municipality. Demographic data of these 15,000 Italian families derived from the 2001 census were fed into a computer. A quota sampling was performed by geographical region, type of municipality, age group, gender, educational level and occupation and controlled by the computer during the whole survey, closing each quota after completion. The administration of the interview took on average 13 minutes.

## Results

Table 1 shows the demographic characteristics of the sample. The 1001 participants had a mean age of 48.1 years (SD = 17.7), were 51.9% female and 42.7% had at least high school diploma. The response rate was 60%.

**Table 1:** Demographic characteristics of the sample.

SEX	N	%
Male	481	48.1
Female	520	51.9
AGE		
<31 years	159	15.9
31–40 years	234	23.4
41–50 years	156	15.6
51–60 years	190	18.9
61–70 years	144	14.4
>70 years	123	12.3
EDUCATION		
University degree	102	10.2
University (no degree)	17	1.7
High school diploma	308	30.8
High school (no diploma)	56	5.6
Secondary school diploma	259	25.9
Secondary school (no diploma)	43	4.3
Primary school diploma	197	19.7
Primary school (no diploma)	14	1.4
No schooling	4	.4
AREA OF RESIDENCE		
North-western Italy	272	27.2
North-eastern Italy	191	19.0
Central Italy	196	19.5
Southern Italy	232	23.2
Main islands	111	11.1
CITY OF RESIDENCE		
Provincial/regional capital	297	29.7
Other	704	70.3
TOTAL	1001	100.0

### Knowledge and Attitudes Towards Depression

This topic was assessed with three questions, the first asking about awareness of depression, the second listing 4 definitions of depression and assessing the respondent’s agreement with each of them, and a third asking to estimate the number of people suffering from depression at least once in their lifetime prevalence of depression. Virtually all the sample (98%) reported that they aware of depression. One third was unable to estimate the percentage of people suffering (or who had suffered in the lifetime) from depression. Of those providing an estimate, the large majority (93%) indicated a percentage ≥15%. Only 7% of respondents reported that depression was affecting less than 15% of the population.

The estimated prevalence of depression was significantly higher among women (t-test = 4.76, df = 720, p<0.001) and those who had a personal (direct or indirect) experience with depression (t-test = 4.14, df = 720, p<0.001) and did not vary significantly by age group.

When respondents were asked to endorse the extent to which they agreed with four different definitions of depression, for the large majority, depression was “a commonplace illness” (77%) or “a state of excessive anxiety” (72%). On the other hand, more than half of the respondents viewed depression as a “personal weakness” (55%) or “a mental illness” (58%).

### Personal Experience with Depression

Experience with depression was explored with 4 yes/no questions assessing whether the respondent, a respondent’s friend or family member had suffered from depression and whether the respondent was a health professional providing treatment for depression. Sixty-two percent of the sample had experienced depression, either directly or indirectly, whereas 38% of the population reported no such experience. More specifically, one in five participants reported personal experience with depression, almost half of the population (44%) had a friend who experienced depression, while about one third (33%) reported that a member of their family had suffered from the disease; those who experienced depression from a professional standpoint (i.e. as care providers) were a small minority (12%).

**Figure 1:**
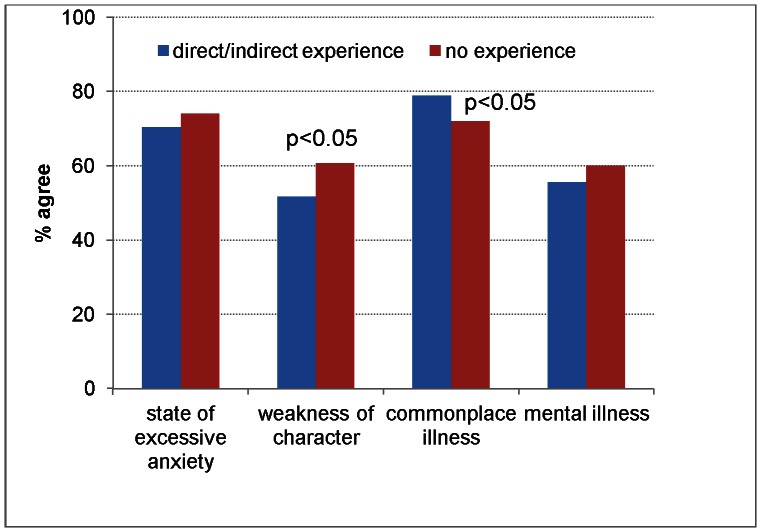
Agreement with 4 definitions of depression in respondents with or without a personal experience of depression.

Those who reported direct experience were significantly more likely to consider depression as a commonplace illness, whereas those who never experienced depression viewed it as a personal weakness rather than a full-blown disorder (fig. 1).

### Stigmatization Processes

Attitudes towards people suffering from depression were surveyed with five items (table 2).

**Table 2:** Stigmatization processes.

	% agreement
People suffering from depression tend to withhold their condition	75
People suffering from depression can overcome their problem by themselves if they want	31
Employers should not hire people suffering from depression	30
People suffering from depression are dangerous to others	27
Coming into contact with people suffering from depression may expose to the risk of contracting the same illness	16

The prevailing belief was that “sufferers from depression tend to withhold their condition” (75%).

On the other hand, the least shared view of depression (16%) was that “coming into contact with people suffering from depression may expose to the risk of contracting the same illness”. There was no particular prejudice about having social contacts with people experiencing depression (and about their right to have a social life), but there was a marked tendency to consider depression as a very personal/private experience.

About one third of respondents (31%) thought that people with depression “can solve their problem by themselves if they want”), while according to one fourth of the respondents (27%) people experiencing depression “are dangerous to others”. In other words, a minority of the sample still viewed depression as a “socially dangerous” illness, or a condition that could be managed without recourse to external help.

### Specific Nature of Depression and Illness-related Problems

In this section, the main symptoms of depression were listed and respondents were asked to indicate whether these symptoms were characterizing a person with depression According to 9 out of 10 respondents, people with depression experience a state of deep sadness. Other commonly endorsed illness-related symptoms were feelings of worthlessness (82%), constant fatigue (77%), suicidal ideation (77%), poor physical health or fatigue (75%).

Other symptoms that respondents strongly associated with depression were thought and concentration problems (71%) and guilt feelings (70%). A condition of irritability, anger or nervousness was less strongly, though rather frequently, associated with depression. On the other hand, loss of appetite was associated with depression by slightly more than half of respondents (56%).

### Causal Beliefs about Depression

Participants were asked to rate the relative likelihood of some particular causes of depression in comparison to other potential causes. Among the “psychological” (stress, traumatic events, divorce/separation from spouse) or “biological” (predisposition, physical illnesses, genetic heredity) options listed as possible causes, the most frequent cause endorsed is that depression can be the consequence of a stressful situation or a traumatic event (see Table 3).

**Table 3:** Causes of depression.

	% likely
Recent traumatic events (job loss etc.)	90
Stressful situations (family disagreements, poverty)	87
Serious physical illness	86
Divorce, end of relationship	84
Recent death of a friend or a close relative	82
Problems during childhood (abuse, early loss of a parent)	73
Personal weakness, nervousness	66
An illness of the brain	65
Recent pregnancy	65
Menopause	52
Having parents or grandparents suffering from depression	47

Many respondents associated depression with a specific life stressor such as serious illness (86%), divorce or separation (84%), mourning for a friend or close family member (82%).

After the so-called “psychological” causes (i.e. psychological reactions to life stressors), respondents mentioned a number of “predisposing” factors rooted in each individual: unsolved problems emerging during childhood (73%), personal weakness or nervousness (66%), previous brain diseases or other brain problems (65%), hereditary factors, i.e. parents or grandparents suffering from depression (47%).

Abouth two thirds of the total population (65%) indicated recent pregnancy as a possibile cause of depression; half of the sample (52%) associated depression to menopause.

### Choice of Treatments

Participants were asked to select from a list the person (either a health professional or a lay person) they would recommend to a friend or a relative seeking help for depression.

**Figure 2:**
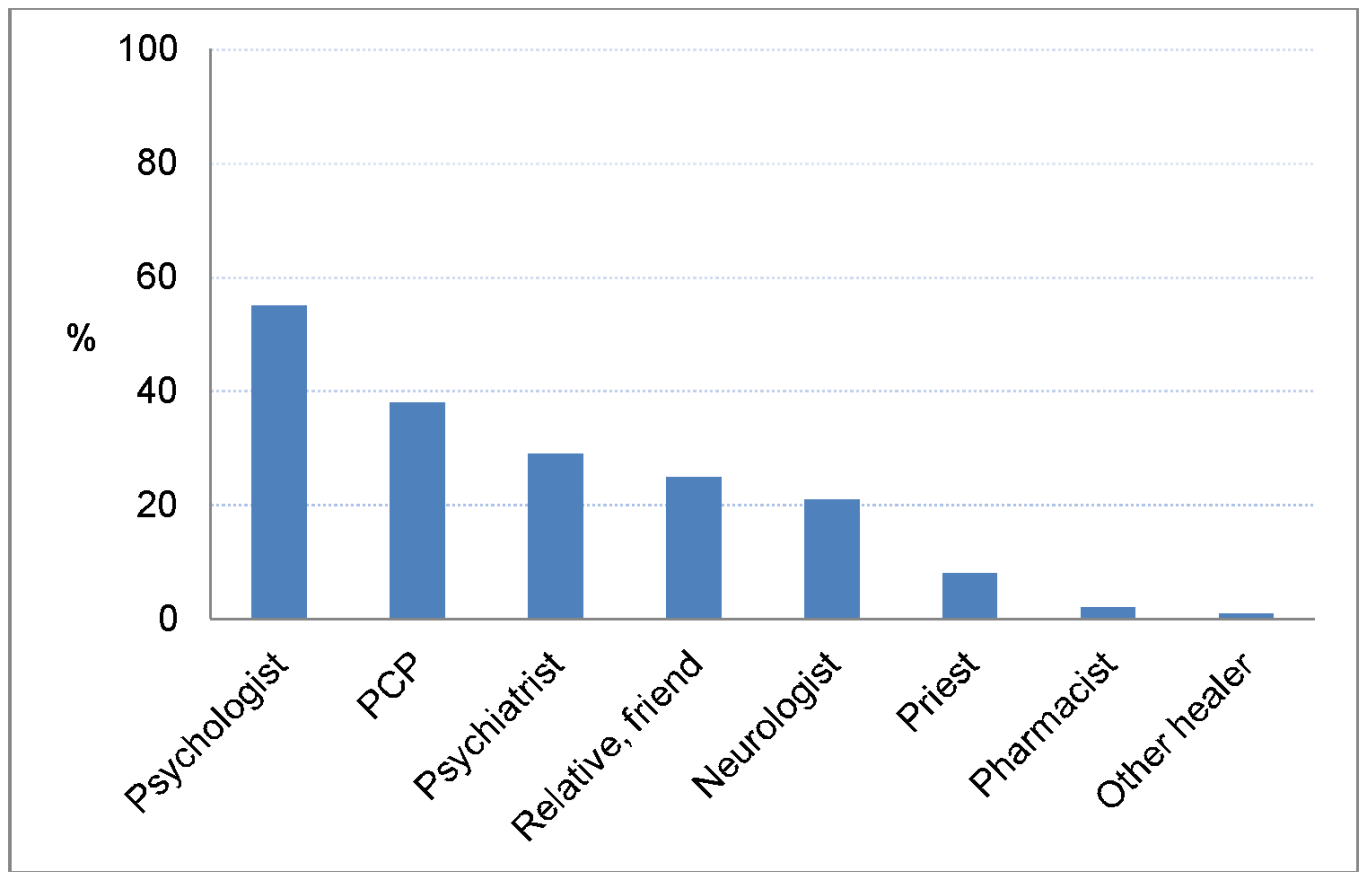
Source of help for depression.

Virtually all (99%) thought that the best way to recover from depression consisted in seeking help from outside: among those who shared this view, the best providers of help were thought to be psychologists (55%), primary care physicians (PCPs, 38%), psychiatrists (29%), and neurologists (21% ) (Figure 2).

Besides health care professionals, about 1/3 of respondents also mentioned as useful source of help a number of subjects who could offer some sort of emotional support: friends, relatives, colleagues, and priests. Pharmacists and healers practising alternative medicine were at the bottom of the list of possible helpers (1–2% of responses).

In a multiple logistic regression model, the likehood of advising a depressed friend or a relative to seek help from a psychologist was significantly higher in the age group 41–50 years than in elderly people (> = 70 years) (OR = 1.8, 95% CI 1.1–3.0), as well as among university graduates compared with people with up to 5 years of education OR = 1.9, 95% CI 1.1–3.1).

Then, participants were asked to report their agreement with three statements on the role of primary care physicians as help for depression.

About 58% of the respondents reported that people suffering from depression would feel quite embarassed in discussing their problem with a PCP; on the other hand, respondents did not seem to have a clear idea of how PCPs deal with with people suffering from depression. While 54% of the agreed in considering family doctors as fully capable to understand and help patients suffering from depression, an almost matching percentage believed that they are too busy to take care of depressed patients (see Table 4).

**Table 4:** Views on primary care providers.

	% agreement
People suffering from depression would be embarrassed in discussing the problem with their family doctor	58
Primary care doctors are fully capable to understand and help patients suffering from depression	54
Family doctors are too busy to take care of depressed patients	52

In a multiple logistic regression model, the percentage of those feeling that seeking help from a family doctor for depression would be embarrassing was significantly lower among those who had a direct experience (OR = 0.59, 95% CI 0.42–0.83) and was unrelated with gender, age or education.

### Treatment Preferences

Treatment preferences were assessed with a set of statements about drug medications, psychotherapy and alternative treatments.

About half of the respondents (53%) agreed on the importance of pharmacological treatment. However, many of them also seemed convinced that antidepressant drugs are addictive (64%) and may cause serious side effects (55%).

60% of the respondents indicated antidepressant as the best treatment option; 56% vitamins and tonics; 48% tranquillizers, anxyolitics and sleeping pills.

High preference rates for vitamins are consistent with widespread fears about possible addictiveness or side effects of antidepressants.

The percentage of those thinking that depression should be treated with vitamins or tonics was similar across all subgroups, while recourse to antidepressants was suggested significantly more frequently by respondents with higher education (high-school or university graduates), average income (as distinct from lower-income categories), and direct or indirect experience of depression.

Advisability of psychotherapeutic treatment (clinical encounters and/or psychotherapy) in the care of depression is widely recognized: more than 80% (with a similar distribution across all subgroups) agreed that depression should be treated with support encounters aimed at symptom management; 70% considered long-term psychotherapy as a possible treatment option, but only 30% of the sample argue for brief psychotherapy (less than 10 encounters).

Among the different forms of “alternative”, non-drug treatment of depression (see table 5), respondents indicated support from loved ones: nine people out of ten (across all subgroups) seem convinced that stronger support from relatives and friends is necessary in order to overcome depression.

**Table 5:** Views on coping strategies and other forms of treatment.

	% agreement
Depression can be overcome with stronger help from relatives and friends	89
Physical activity (e.g. practising sports, walking) can be useful in overcoming depression	83
Depression can be overcome by solving one’s social problems (unemployment, poverty, family problems)	81
Depression can be overcome with some training in relaxation and stress management techniques	62
Depression can be overcome by oneself, without referring to a specialist	20
Depression can be cured with hypnosis or acupuncture	17
Depression can be overcome by drinking alcohol	4
Depression can be overcome with narcotic drugs	4

Exercise and psychological well-being were also considered essential: more intense physical activity (83%), overcoming of one’s social problems (81%), attending relaxation or stress management classes (62%).

Consistent with above-mentioned views on the perceived usefulness of professional help and psychotherapy, 80% of the sample agreed that depression cannot be overcome without help from a health care professional.

A small minority (17% of the sample) thought that depression could be trated with hypnosis or acupuncture, and even fewer respondents (4%) endorsed recourse to alcohol or narcotic drugs as possible coping strategies.

## Discussion

The results of the present survey indicate that depression is a common experience, affecting directly or indirectly 62% of the people interviewed. Among those reporting this experience, depression was more frequently defined as a commonplace illness than as a weakness of character.

Life stressors or physical strains were the most endorsed causes of depression. Consistent with this view, psychologists were often indicated as the best source of professional help. Half of the sample agreed that depression should be pharmacologically treated, but drugs were often seen as addictive, and liable to produce side effects. Seeking help from a primary care physician was considered embarrassing; furthermore, many people thought that PCPs are too busy to treat patients suffering from depression.

Our results suggest that the Italians are aware of depression and that a direct or indirect experience favours less stigmatizing attitudes. Although one third of the sample was unable to estimate the lifetime prevalence of depression, two thirds indicated that depression is a non-marginal problem affecting more than 15% of the population.A comparable tendency to overestimate the prevalence of depression has been reported by other studies [24], [25], but the propensity seems higher in the Italian sample.

A non-negligible part of the nationwide sample (about one third of total respondents) expressed various prejudices towards depression and people with depression: “a dangerous illness” that could be overcome “with some willpower”, a “disabling condition” for one’s job career. Another widespread belief (75% of the sample) is that people suffering from depression should avoid talking about their problem, i.e. depression should be experienced in solitude. The belief in the helpfulness of self-reliance in coping with depression is consistent with the findings from an Australian survey [26].

Stigmatisation processes resulted stronger in the present study than in other countries (e.g. in Canada: [27]).

However, the percentage of responders endorsing that people suffering from depression are dangerous to the others is higher in the United States [28] (33%) and in Brazil [29] (56%) than in the present study (27%).

The explanation of this finding is that in Italy the onset of depression is generally ascribed to stressful situations (nonspecific stress, and/or post-traumatic stress ensuing painful events such as bereavement or divorce). while hereditary causes (such as brain diseases, parents suffering from depression, etc.) are deemed less important. Thus, depression appears as a predictable reaction to a stressful event. Comparable views were expressed in an English survey [17] and in a Swedish study on primary care patients [30].

The psycho-social view of depression is consistent with responses concerning the choice of an appropriate source for help.

Eighty percent agree that people experiencing depression should seek help from a “qualified” professional (i.e. a psychologist, a PCP or a neurologist); on the other hand, 3 in 5 are convinced that discussing the problem with one’s family doctor would be quite embarrassing. PCPs are thought capable to supply adequate help and understanding, but at the same time too overburdened with patients to treat depression with the required amount of attention and competence, in line with a survey carried out in Great Britain [31]. PCP and psychiatrists are less frequently suggested as possible sources of help by Italian respondents than by participants to other surveys in different countries such as Russia, Slovakia, Germany [32] or Australia [25], [33]. One of the most frequent perceived barriers to disclosure of depressive symptoms to PCPs in a US study is the concern that the physician would recommend antidepressants [34].

In Italy, as in Spain [24], Austria [35], and Brazil [29] psychologists are generally seen as first-choice help for depression problems: discussing depression with a psychologist rather than with one’s family doctor is perceived as less awkward.

As to pharmacological therapy, fear of addictive effects is reported as the main cause for concern, in line with a Turkish study [36]. According to respondents, depression is better cured with vitamins (seen as free from side effects) than with potentially harmful antidepressants. Tranquillizers, anxyolitics and sleeping tablets are widely mentioned among less useful (and more health-damaging) options in depression care. Potential benefits of vitamins compared with antidepressants are likewise stressed by Australian respondents [33].

Feelings about psychosocial treatments are generally more positive: specifically, support encounters for symptom management are considered the best therapeutic approach to depression treatment (85% of favourable responses). One third of the sample believes that depression should be treated with long-term psychotherapy, in line with results from other surveys [14], [33], [36].

As concerns alternative forms of treatment, respondents emphasized the potential benefits of support from loved ones (62%), better care of psychophysical well-being (89%) through more intense physical activity, overcoming one’s social problems, and training in relaxation and stress management techniques. Twenty percent think that depression should be under one’s control, without recourse to a specialist; the percentage of respondents sharing this view is higher than that reported in the only comparable study (12% of population, see Jorm *et al*., [33).

### Limitations

The results of this study should be interpreted keeping in mind some limitations that are inherent in the telephone interviews. First, people agreeing to be interviewed may have different characteristics from people who refuse to be interviewed. Although our response rate was 60%, it was higher than that reported in a recent Australian surveys involving people aged 15–25 years (47.9%) [23].

Second our landline-based estimates are biased by the absence of cell-phone only population, that is continuously growing over time.Still, this limitation is mitigated by the fact that in Italy the very large majority of families had a landline telephone when the interview was carried out. The interview has no established validity and reliability, and further investigation is needed to assess its psychometric properties.

### Conclusions

Overall, our findings indicate that depression is seen as a reaction to a significant life event that should be overcome with the support of significant others, the help of health professionals (mainly psychologists) and a variety of alternative strategies. However, there are still barriers to the disclosure of depressive symptoms to PCPs, linked to the belief that they are too busy, and concerns about antidepressants, that are seen as potentially harmful and addictive. In the presence of a gap between people’s beliefs and what health professionals consider appropriate for the treatment of depression, a “shared decision making” approach to treatment selection should be adopted taking into account the patients’ preference for psychological interventions and ensure active compliance with effective treatments.

## Supporting Information

Appendix S1 **Questionnaire about people’s views and preferences on depression treatment.**
(DOC)
