# Supplementary material for: Public Beliefs and Attitudes towards Depression in Italy: A National Survey
Source: PLoS One. 2013 May 20;8(5):e63806. doi: 10.1371/journal.pone.0063806 (PMC3659050; doi:10.1371/journal.pone.0063806)
Supplement: Appendix S1 — Questionnaire about people’s views and preferences on depression treatment. (DOC) [file pone.0063806.s001.doc]

| *STRICTLY CONFIDENTIAL* April 2009 | 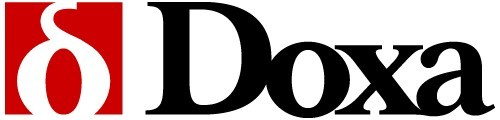  ISTITUTO PER LE RICERCHE STATISTICHE E L'ANALISI DELL'OPINIONE PUBBLICA  MILANO - Via B. Panizza, 7 – Tel. (02) 48.19.32.76 - Milano S. 10068/C – People’s views and preferenceson depression treatment(Individuals 15 years old and over) | *INTERVIEWER: __________*  *INTERVIEW N.: _____*  *DATE: _____________*  *LOCATION: __________* |
| --- | --- | --- |

Good morning/afternoon/evening, I am calling from Doxa Institute. We are conducting a survey on behalf of the Study and Research Centre in Psychiatry of Turin, that carries out the survey funded by the Ministry of Health.

The survey is of national scope and its purpose is to collect people’s opinions and preferences on depression treatment, in order to improve the provision of health care services.

May I talk with… (ask for a person with gender, age, school degree and working status as required by the sample’s stratification)?

May I ask you some questions for an interview that will last about 10-12 minutes?

**PART ONE: SOCIO-DEMOGRAPHIC CHARACTERISTICS**

D1: Age

| 1. How old are you? |  |
| --- | --- |

D2: Gender *[do not ask]*

|  | **Male** | **Female** |
| --- | --- | --- |
| 1. Gender |  |  |

D3: Marital status

|  | **Not married and not cohabiting** | **Married or cohabiting** | **Separated or divorced** | **Widowed** |
| --- | --- | --- | --- | --- |
| 1. What is your marital status? |  |  |  |  |

D4: Nationality

| 1. What is your nationality? _________________________ |
| --- |

D5: School degree

|  | **Primary school degree** | **Secondary school degree** | **High school degree** | **University degree** |
| --- | --- | --- | --- | --- |
| 1. What is your school degree? |  |  |  |  |

D6: Working status

|  | **Working** | **Not working** |
| --- | --- | --- |
| What is your working status? |  |  |

**PART TWO: ATTITUDES ON DEPRESSION**

D7: Problem awareness

|  | **Yes** | **No** |
| --- | --- | --- |
| 1. Have you ever heard of depression? |  |  |

D8: Definition of depression

| Now, I’m going to read you some statements other respondents made about depression. For each of them, tell me please whether you agree strongly, fairly, little or not at all.  **(Read the statements one by one – rotate – one answer per statement)** | **Strongly agree** | **Fairly agree** | **Little agree** | **Not at all agree** | **(don’t know)** |
| --- | --- | --- | --- | --- | --- |
| 1. Depression is a state of excessive concern |  |  |  |  |  |
| 1. Depression is weakness of character |  |  |  |  |  |
| 1. Depression is a medical condition like other illnesses |  |  |  |  |  |
| 1. Depression is a mental illness |  |  |  |  |  |

D9: Opinion on prevalence of depression

|  | **%** | **don’t know** |
| --- | --- | --- |
| 1. Do you have an idea, even vague, of how many people have suffered from depression in Italy at least once in their life? Can you give a percentage? |  |  |

D10: Stigmatization process

| Now, I’m going to read you some statements other respondents made about people suffering from depression. For each of them, tell me please whether you agree strongly, fairly, little or not at all with it.  **(Read the statements one by one – rotate – one answer per statement)** | **Strongly agree** | **Fairly agree** | **Little agree** | **Not at all agree** | **(don’t know)** |
| --- | --- | --- | --- | --- | --- |
| 1. People suffering from depression can solve their problem by themselves, if they want to |  |  |  |  |  |
| 1. It is better to avoid visiting people suffering from depression if you do not want to be depressed, too |  |  |  |  |  |
| 1. People suffering from depression are dangerous to the others |  |  |  |  |  |
| 1. People suffering from depression tend not to say it |  |  |  |  |  |
| 1. An employer would rather not hire people suffering from depression |  |  |  |  |  |

D11: Problems and experiences of depression

| Do you think that people suffering from depression experience the following moods and conditions more often than people who do not suffer from depression?  **(Read the statements one by one – rotate – one answer per statement)** | **Yes** | **No** | **Maybe (or don’t know)**  **ONLY WHEN SPONTANEOUS** |
| --- | --- | --- | --- |
| 1. Feeling sad, feeling down |  |  |  |
| 1. Feeling always tired |  |  |  |
| 1. Having no appetite |  |  |  |
| 1. Thinking to be worth nothing |  |  |  |
| 1. Feeling weak and sick |  |  |  |
| 1. Feeling irritable, angry or nervous |  |  |  |
| 1. Feeling guilty |  |  |  |
| 1. Finding it hard to concentrate or think |  |  |  |
| 1. Having suicidal thoughts |  |  |  |

D12: Causes of depression

| Now, I’m going to read you some situations and events. For each of them, tell me please which one is likely to cause depression.  **(Read the statements one by one – rotate – one answer per statement)** | **Very likely** | **Rather likely** | **Little likely** | **Not at all likely** | **don’t know** |
| --- | --- | --- | --- | --- | --- |
| 1. A brain illness |  |  |  |  |  |
| 1. Stress situations such as family quarrels, financial difficulties |  |  |  |  |  |
| 1. The recent passing away of a close friend or relative |  |  |  |  |  |
| 1. A traumatic recent event such as losing the job |  |  |  |  |  |
| 1. A divorce or the end of a relationship |  |  |  |  |  |
| 1. Childhood problems (i.e. being abused or having lost a parent as little child) |  |  |  |  |  |
| 1. To have a weak or nervous character |  |  |  |  |  |
| 1. To have parents or grandparents suffering/having suffered from depression |  |  |  |  |  |
| 1. To have a sever illness |  |  |  |  |  |
| 1. To have just had a pregnancy |  |  |  |  |  |
| 1. To be in menopause |  |  |  |  |  |

**PART THREE: CHOICE OF TREATMENT**

D13: Choice of the most proper person to be helped by

| 1. Now, I’m going to read you a list of people that can be helpful in dealing with depression: GP, pharmacist, psychiatrist, neurologist, psychologist, relative, friend or colleague, priest and healer. If a close relative or friend suffered from depression, who would you suggest to consult?   **(Read - rotate - more answers allowed)**   1. And among these people who would you choose as first?   **(read the answers to Q. 13a - one answer only)** |  |
| --- | --- |
| 1. The PCP |  |
| 1. A pharmacist |  |
| 1. A psychiatrist |  |
| 1. A neurologist |  |
| 1. A psychologist |  |
| 1. A relative, a friend or a colleague |  |
| 1. A priest |  |
| 1. A healer (e.g. expert in alternative medicine, pranotherapist, magician, etc.) |  |
| 1. Other (please, specify) _____________________________________ |  |

D14: Views on the primary care physician (PCP)

| Now, I’m going to read you some statements other respondents made about the PCP as the suitable professional to treat depression. For each of them, tell me please whether you agree strongly, fairly, little or not at all.  **(Read the statements one by one – rotate – one answer per statement)** | **Strongly agree** | **Fairly agree** | **Little agree** | **Not at all agree** | **(don’t know)** |
| --- | --- | --- | --- | --- | --- |
| 1. People suffering from depression are embarrassed to go to their PCP |  |  |  |  |  |
| 1. PCPs usually understand and help properly people suffering from depression |  |  |  |  |  |
| 1. PCPs are too busy to treat patients suffering from depression |  |  |  |  |  |

D15: Views on drugs

| Now, I’m going to read you some statements other respondents made about drugs as the method to treat depression. For each of them, please tell me whether you agree strongly, fairly, little or not at all.  **(Read the statements one by one - one answer per statement)** | **Strongly agree** | **Fairly agree** | **Little agree** | **Not at all agree** | **(don’t know)** |
| --- | --- | --- | --- | --- | --- |
| 1. Depression is a disease that needs being treated with drugs |  |  |  |  |  |
| 1. The drugs used to treat depression are addictive |  |  |  |  |  |
| 1. The drugs used to treat depression have severe side effects |  |  |  |  |  |

D17: Psychotherapeutic treatments

| Now, I’m going to read you some statements other respondents made about psychotherapy as the method to treat depression. For each of them, tell me please whether you agree strongly, fairly, little or not at all.  **(Read the statements one by one - rotate - one answer per statement)** | **Strongly agree** | **Fairly agree** | **Little agree** | **Not at all agree** | **(don’t know)** |
| --- | --- | --- | --- | --- | --- |
| 1. Depression is a disease that needs to be treated with counselling in order to deal with symptoms |  |  |  |  |  |
| 1. Depression is a disease that needs to be treated with a short-term psychotherapeutic treatment (less than 10 sessions) |  |  |  |  |  |
| 1. Depression is a disease that needs to be treated with a long-term psychotherapeutic treatment |  |  |  |  |  |

D18: How to deal with the problem

| Now, I’m going to read you some statements other respondents made about strategies to deal with depression. For each of them, tell me please whether you agree strongly, fairly, little or not at all.  **(Read the statements one by one - rotate - one answer per statement)** | **Strongly agree** | **Fairly agree** | **Little agree** | **Not at all agree** | **(don’t know)** |
| --- | --- | --- | --- | --- | --- |
| 1. Depression can be solved alone, without going to a specialist |  |  |  |  |  |
| 1. Depression can be solved by resolving one’s social problems (unemployment, poverty, family problems) |  |  |  |  |  |
| 1. Depression can be solved by receiving more support by family and friends |  |  |  |  |  |
| 1. Depression can be solved by becoming physically more active (e.g. practicing more sport, walking more) |  |  |  |  |  |
| 1. Depression can be solved by attending a relaxation course, to manage the stress |  |  |  |  |  |
| 1. Depression can be solved by drinking alcohol |  |  |  |  |  |
| 1. Depression can be solved by using drugs |  |  |  |  |  |
| 1. Depression can be solved by undertaking hypnosis or acupuncture treatments |  |  |  |  |  |

D19: Usefulness of some drugs to treat depression

| I’m going to mention you some categories of drugs. For each of them, tell me please whether you rate them necessary, useful, useless or harmful to treat depression.  **(Read the categories one by one - rotate - one answer per category)** | **Necessary** | **Useful** | **Useless** | **Harmful** | **(don’t know)** |
| --- | --- | --- | --- | --- | --- |
| 1. Vitamins or tonics |  |  |  |  |  |
| 1. Antidepressants |  |  |  |  |  |
| 1. Tranquillizers, anxiety or sleeping pills |  |  |  |  |  |

**PART FOUR: EXPERIENCE OF DEPRESSION TREATMENT**

D20: Personal experience with depression

| Concluding, some questions about your personal experience with depression | **yes** | **no** | **(don’t know/missing)** |
| --- | --- | --- | --- |
| 1. Among your friends, has anybody ever suffered from depression? |  |  |  |
| 1. In your family has anybody ever suffered from depression? |  |  |  |
| 1. Have you ever suffered from depression? |  |  |  |
| 1. In your life, have you ever tried to provide professional services or treatments to people suffering from depression? |  |  |  |

The interview is over

Thank you for you help and the time you spent for this survey. I would like to remind you that I’m calling from Doxa Institute on behalf of the Study and Research Centre in Psychiatry of Turin. I would also like to remind you that, according to the Italian Privacy Law, any information and opinion you gave me will be used for this research purpose only.
